# Knowing in Nurses’ Belief and Attitude about Patient Activation: A Validation of the Korean Clinician Support for Patient Activation Measure Using Rasch Analysis

**DOI:** 10.3390/healthcare8040571

**Published:** 2020-12-17

**Authors:** Suhyeon Choi, Yun Hee Ham, Kihye Han, Eunjung Ryu

**Affiliations:** 1 Graduate School, Chung-Ang University, Seoul 06974, Korea; suhyeonch@gmail.com; 2 Department of Nursing, Samsung Medical Center and Graduate School, Chung-Ang University, Seoul 06351, Korea; yunhee.ham@samsung.com; 3 Department of Nursing, Chung-Ang University, Seoul 06974, Korea; hankihye@cau.ac.kr

**Keywords:** self-management, patient activation, nurse, attitude

## Abstract

Background: Patient engagement is considered a critical factor in improving healthcare delivery. This study aimed to test the Korean version of the Clinician Support for Patient Activation Measure (CS-PAM) using Rasch analysis, and to explore nurses’ beliefs about patient self-management. Methods: A cross-sectional, exploratory study design was employed. The staff nurses who were recruited from six hospitals were requested to complete the Korean CS-PAM. Their responses were subsequently subjected to Rasch analysis to validate the Korean CS-PAM. The CS-PAM was paraphrased into Korean using the standardized forward–backward translation method. Results: The internal consistency of the scale had good Cronbach’s alpha value. For all items, the infit and outfit statistics fell well within the acceptable range of 0.5–1.5. This measure formed a unidimensional Guttman-like scale that explained 54.7% of the variance. Conclusions: The Korean version of the CS-PAM showed good psychometric properties and appeared to be consistent with the meaning of the original CS-PAM. However, the items have a somewhat different ranking order when compared to the English and Dutch versions. The instrument might be useful for identifying the supportive beliefs and attitudes of nurses or healthcare providers in order to improve patient activation in healthcare.

## 1. Introduction

The perspective of patient care has gradually shifted from a focus on treating the disease to caring for the patient holistically. This change has been attributed mainly to the growing number of chronic diseases [1]. Patients with chronic illness must concentrate on taking their medication, making lifestyle changes, and dealing with symptoms [2,3], but generally face considerable difficulty in managing their chronic conditions because of the nature of the long-term treatment. However, the patient’s involvement in their healthcare is critical for generating innovative actions in the complex context of healthcare delivery [4]. Patient self-management is particularly essential for successful health management, and active patients tend to show improvement in their self-management behavior [2,5].

In Asian countries, family members have traditionally been significant participants in supporting patient care; however, as families in these countries have begun to shrink, coupled with the increase in the length of the disease period, the aging population, etc., the role of the primary care provider is becoming increasingly important. Nurses are particularly crucial for improving the partnership between patients and other health professionals. If nurses convince patients of the importance of self-management, they may be able to better support patients and improve their healthcare outcomes. Indeed, studies have demonstrated the importance of primary care providers’ support for patient self-management [6,7].

However, even if nurses do perceive the importance of patient self-management, they often find it challenging to engage in patient consultations due to their high workloads and the limited time for that purpose [8,9]. Researchers have suggested providing clinicians with adequate training in supporting patients and developed the Clinician Support for Patient Activation Measure (CS-PAM) for evaluating physicians’ support of patients’ self-management [1].

Many patients have conventionally undertaken a passive attitude toward healthcare providers and their care, reflecting general social norms governing the professional–patient relationship [10]. Hage and Marwell [11] presented a programmatic approach to the development of a theory of role relationships, in which they suggest that the integration of a link refers to the articulation of that relationship vis-à-vis other relationships. Many nursing models have emphasized the importance of relationships regardless of their philosophical underpinnings. Notably, the relationship between nurse and patient is crucial to successful care outcomes. In patient-centered nursing, having positive beliefs and attitudes toward patient self-management is a step in the direction of patient activation.

To our knowledge, there is no Korean questionnaire designed to assess the level of nurses’ beliefs and attitudes in relation to patient activation. Most outcome measures used in healthcare focus on attributes that are not directly measurable, such as pain, self-esteem, or quality of life. These measures give a manifest score of the construct being measured. Rasch analysis has been used to aid in the construction and validation of these questionnaires and to assess human performance, attitude, and perceptions [12]. The Rasch model also provides a means of assessing a range of additional measurement properties, increasing the information available on a scale’s performance [12,13].

Therefore, the CS-PAM was translated and required further evaluation as to whether it has the ability to assess the level of nurses’ beliefs and attitudes. The purpose of this study was to analyze the psychometric properties via the Korean version of the CS-PAM (Korean CS-PAM) with Rasch analysis, and to assess whether there are differences in the beliefs of Korean nurses about patient’s activation behaviors by types of hospital and nurse positions.

## 2. Materials and Methods

### 2.1. Design and Participant

A cross-sectional design was employed, with a total of 1430 nurses recruited from 6 hospitals located in South Korea. The 6 hospitals were conveniently selected based on bed size (1 hospital with <500 beds, 2 with 500–800 beds, and 3 with >800 beds), type of hospital (3 general hospitals and 3 tertiary hospitals), and location (2 in Seoul, 4 outside of Seoul). Participants returned the questionnaire by placing it in a sealed envelope to remain anonymous. Data collection took place from April 2017 to March 2018. The response rate was 91%, with 1302 of the 1430 questionnaires being returned. 1204 of them competed Korean CS-PAM.

The three types of nurses included staff nurses, advanced practice nurses, specialized nurses (who assist physicians in specialized units and act as ‘bridge’ personnel between doctors and nurses, such as in operation or wound care) and unit managers.

### 2.2. Measures

The 13-item CS-PAM measures nurses’ beliefs and attitudes toward patient self-management, indicating the degree of importance of patient behavior in the nurse’s view [1]. The CS-PAM items are prefaced with the following: “As a clinician (or nurse), how important is it to you that patients with long-term conditions are able to…” Nurses answer each item by using a 4-point response scale ranging from 1 (not important) to 4 (extremely important). Higher scores indicate a stronger belief in patients’ self-management. The total score was recalculated to range from 0 to 100 using Rasch methods.

The CS-PAM was translated and adapted using the systematic approach proposed by the World Health Organization instructions, which was also used in translating the PAM [14,15]. This approach had the following steps: (1) Two bilingual expert researchers did independent forward translations. (2) Two bilingual nonhealthcare professionals did back translations. (3) The language and similarity of interpretability of the original and back-translated versions were compared by two native English-speaking healthcare professionals and two nonhealthcare professionals. To ensure semantic equivalence and content validity, they ranked each item in the original and translated versions in terms of comparability of language and similarity of interpretability using a Likert-type Scale ranging from 1 (extremely comparable/extremely similar) to 7 (not at all comparable/not at all similar) [16,17]. (4) A pre-test was conducted among 15 Korean nurses for readability and acceptability.

### 2.3. Data Analysis

In the Rasch analysis, data from 135 (10.4%) individuals identified as misfits (response aberrancy) were excluded, so the analysis was performed on the data of the 1069 nurses. The missing completely at random (MCAR) test was undertaken on the full dataset. The calculation of all variables for each case found a random distribution of missing data (Little’s MCAR test: χ^2^ = 121.296, df = 120, *p* = 0.45), indicating about a 10% missing data value was adopted [18].

The psychometric properties of the rating scale were investigated using Rasch analysis measurement. The Winsteps version 4.0.1 (Dr. Linacre, Chicago, IL, USA) was used for the Rasch analysis. Observed average and outfit mean square values (MNSQ) were used to identify the compatibility of the data with the Rasch model. Descriptive analysis was performed using SPSS Statistics 25 (IBM Inc., Skokie, IL, USA) with a significance level of 0.05.

Step 1: The measurement model calibrated the difficulty of the items in terms of response probabilities. Specifically, it created a measure with a theoretical scoring of 0–100. Item scale locations were transformed from the original logit metric to a user-friendly 0 to 100 score, where 0 was the easiest and 100 was the most severe difficulty. While the parameter allows for a potential range of 0–100, the items in the measure only covered the range of 40–60, meaning that the measure does not capture the lowest or highest ranges of the construct [1]. The item calibrations, which indicate how difficult it is for respondents to endorse or agree with that item, were established separately from the individual respondent scores.

Step 2: Item fit statistics (infit and outfit) describe how accurately or predictably the responses to that item fit the model. In-fit relates to the inlier sensitivity of the item (for items with a difficulty that is close to the person), while outfit relates to the outlier sensitivity (for items with difficulty far from the person). An item was excluded as a misfit if its infit MNSQ did not display within the range of 0.6 to 1.4 [12].

Step 3: Uniform differential item functioning (DIF) was used to explore the stability of item difficulty in the probability of endorsing a specific item related to hospital level and nurse position. The magnitude of DIF was investigated using the Mantel–Haenszel statistics, as reported from the Winsteps program.

Step 4: In Rasch analysis, the items in each domain should assess a single dimension or construct, i.e., unidimensionality [19]. This was assessed by calculating variance explained by measures via a principal component analysis (PCA).

Step 5: We also evaluated the personal reliability or the degree to which an individual’s response pattern conforms to the model. Cronbach’s α was used to evaluate the consistency with which a set of items measures a single construct. Finally, we calculated the item–total correlation and the average inter-item correlations to determine the stability of the measure.

### 2.4. Ethical Consideration

This study was approved by the Ethics Review Board of the university (No. 1041078-201704-HRSB-083-01). After the researchers had explained the purpose and content of the study, informed consent was obtained from all participants.

## 3. Results

### 3.1. Demographic Characteristics of Sample

Most participants were staff nurses (88.6%), followed by unit managers (8.6%) and specialist nurses (2.8%). More than half the staff nurses (69.7%) reported having a bachelor’s degree, and more than half (73.9%) of the unit managers had a master’s degree or higher. Specialists included advanced practice nurses (NP) and nurses who assisted the doctor in special units, such as physician assistants. Among the specialists, 83.3% had a bachelor’s degree or higher. The years of work experience of these groups were 281, 64, and 169.5 months (median), respectively. Table 1 provides detailed demographic characteristics.

### 3.2. Tests of Fit to the Model

The Korean CS-PAM had a calibrated scale range of 40.7–63.30 on the theoretical 0–100 difficulty scale. These item calibrations indicate, in a probabilistic sense, how difficult it is for a respondent to endorse or agree with that item [1]; thus, the Korean CS-PAM appears to cover only the middle range of nursing beliefs about patient activation and does not tap into very high or very low values.

All infit and outfit statistics of the Korean CS-PAM fell well within the 0.5–1.5 acceptable range. Table 2 shows the hierarchical order of the 13 items based on the data of 1157 participants. The results indicated that item 12, “look for trustworthy sources of information about their health and health choices, such as on the web, news stories, or books”, was the most difficult to endorse. Item 1, “are able to take actions that will help prevent or minimize symptoms associated with their health condition”, was the easiest. Both the average measure and the thresholds increased monotonically across the rating scale.

**Figure 1:**
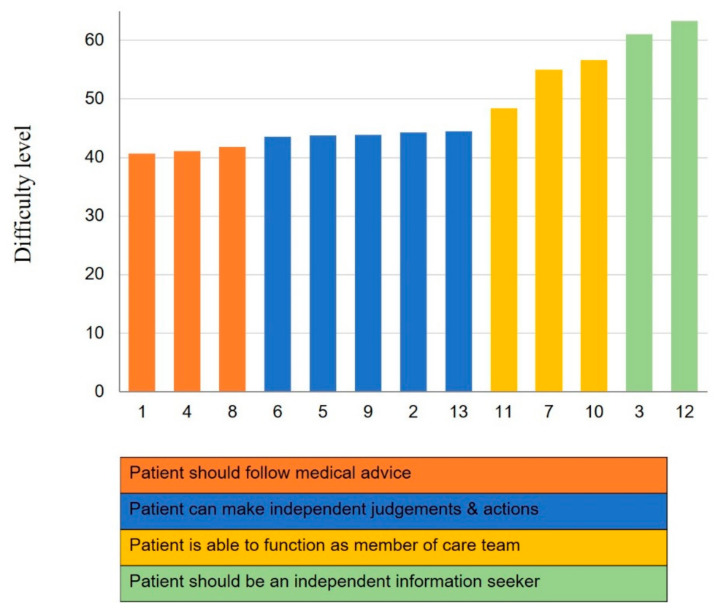
Difficulty structure of the Korean CS-PAM.

As shown in Figure 1, the item hierarchy observed in the scale was confirmed. These results strongly suggest that nurses’ beliefs and attitudes concerning patient activation are developmental. Based on the difficulty structure of the Korean CS-PAM, the items could be categorized into four groups. Group 1 consisted of the relatively ‘easy’ items, which had low calibrations (items 1, 4, and 8). Nurses who endorsed these items believed that patients should follow medical advice. Conversely, Group 4 consisted of the most ‘difficult’ items (i.e., the least likely to be endorsed by nurses) with the highest calibrations (items 3 and 12, which had calibrations of 61.1 and 63.3, respectively). These items concerned beliefs about patients being an independent information seeker.

### 3.3. Analysis of Differential Item Functioning

**Figure 2:**
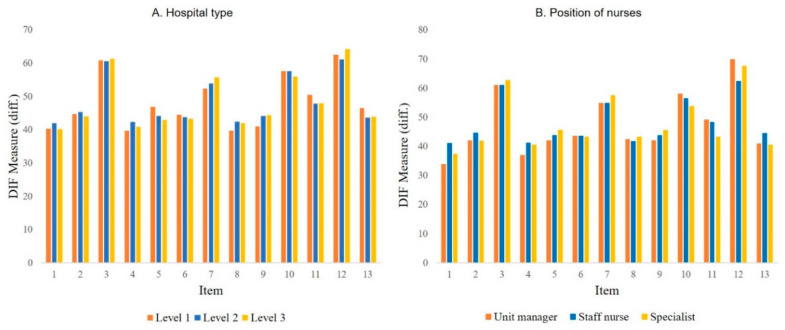
Differential item functioning of the Korean version of the CS-PAM according to hospital type and nurse position. (**A**). Hospital type: item 5 was easier for level 3 than for level 1. (**B**). Nurse position: Unit managers found it easier to answer item 1 than did staff nurses. Staff nurses found it easier to answer item 12 than did the unit managers and specialists. Hospital type. Level 1 = hospital, Level 2 = general hospital, Level 3 = tertiary hospital.

Figure 2 shows the significant DIF comparisons (i.e., whether an item is easier or harder to answer among one group compared to another) for each item on the Korean CS-PAM according to the hospital level and nurse position. All items showed DIF according to the hospital level and nurse position. However, for most items, the difference contrast—which is the difference in the difficulty of the item between groups—was <0.5 logits. The DIF should be at least 0.5 logits to be noticeable [20].

For hospital type, the items showing a significant DIF (i.e., contrast >0.5 logits and probability *p* <0.05) were as follows: item 5 was significantly more difficult for nurses working in hospitals than those working in tertiary hospitals (DIF contrast = 0.57 logits, *p* < 0.01). As for the nurse position, item 1 was more difficult in the staff nurse group compared to the unit manager group (DIF contrast = −1.02 logits, *p* < 0.01). Moreover, the staff nurse group found it significantly easier to answer item 12 than did the unit manager group (DIF contrast = 1.04 logits, *p <* 0.001).

### 3.4. Unidimensionality by Principal Component Analysis (PCA)

A principal component analysis of the residuals was then performed, which yielded a unidimensional structure. The unexplained variance in the first contrast (residual variance) was 1.88, and the variance explained by the first contrast was 8%. This was smaller than the variance explained by the item difficulty, at 23.2%. The model explained a total of 54.7% of the variance in the data.

### 3.5. Reliability

Cronbach’s α coefficient for the Korean CS-PAM was 0.86 (intraclass correlation coefficient = 0.86, 95% CI = 0.84–0.87). A minimum value of 0.7 is required for group use and 0.85 for individual use. Moreover, the overall Rasch personal reliability ranged from 0.77 (real) to 0.79 (model), and the item reliability was 0.99. The separation index for persons was 1.84, and that for items was 15.39, indicating good separation.

## 4. Discussion

This is the first study to investigate the use of CS-PAM in a large sample of Korean nurses. This study was conducted as part of the Shared Governance and Patient Activation Measure Study for Korean nurses. This study investigated the psychometric properties of the Korean CS-PAM using Rasch analysis, which was more appropriate for examining cross-cultural equivalency for psychiatric measures likely to be non-normal [21]. The Rasch analysis of the Korean CS-PAM demonstrated that it was a reliable measure and that inferences made from the item and person measures and the fit information were valid reflections of nurses’ ability, although the ordering of these parameters differed from that of the English and Dutch versions of the CS-PAM.

Healthcare experts, managers, and policymakers are recognizing the importance of a paradigm shift in the planning and delivery of healthcare in favour of having patients take a more active role in the management of their healthcare. In terms of the health service research, they should have a valued voice when it comes to new models of care and new technologies for a broader view of care delivery [22]. Making patients better informed and more directly responsible for their health and care management is pivotal in making healthcare organizations more sustainable at the economic, organizational, and psychological levels [23]. To this end, the Korean CS-PAM score can be used in measuring nurses’ overall levels of endorsement or belief in the importance of patient self-management, as well as their beliefs about the importance of specific patient competency categories [1].

In the context of the CS-PAM, the personal characteristic called ‘ability’ may be conceived as one’s individual beliefs about patient activation; in other words, nurses with higher scores on the CS-PAM will have a higher level of agreement with patient activation. Moreover, the item characteristic of ‘item difficulty’ in the classical Rasch analysis represents in this context the propensity of an item to obtain systematically high or low scores when measuring the latent trait of interest. The item difficult thus reflects the level of relevance each item in the tool has for the measured aspect. Further study could assign each respondent to one of four ‘stages’ of activation, with the first stage corresponding to the lowest level of activation and the fourth stage corresponding to the highest level, according to the CS-PAM scores.

The item calibration scores found in this study were similar to those found in the CS-PAM development study [1], indicating that the difficulty level of the items in the CS-PAM is comparable among clinicians in various Western and Asian countries (i.e., US, UK, the Netherlands, and Korea) [1,3]. Notably, the mean CS-PAM score of Korean nurses was higher than that of clinicians in the US, UK, and Netherlands.

Based on the difficulty structure of the Korean CS-PAM, four groups of items could be established. The ordering of the items within these groups was slightly different from that in the Dutch sample [3], but most items fell with the limits of the original four-item groups described by Hibbard, Collins, Mahoney and Baker [1] (See Appendix A). When instruments are developed using a conceptual hierarchy of items, the empirical ordering produced by the Rasch analysis can be compared with the theoretical ordering, and the result can be treated as evidence of construct validity [19]. Korean nurses appeared to find it easier to endorse items representing how patients should be able to understand the different medical treatment options available for their chronic conditions when compared to clinicians from the US, UK, and Netherlands. Korean nurses also showed greater endorsement of items describing patient behaviors that focused on following medical advice. These behaviors do not reflect the self-management of chronic illness, which requires patients to have more independence [1]. This potential reason could be due to the different professional roles in the sample, the time spent with patients, and perhaps also professional culture [23]. However, this hierarchy in their beliefs and attitudes regarding patient self-management was still similar to that found among the US, UK, and Dutch clinicians [1,3].

Differential item functioning (DIF), or item bias, can also affect the fit of a Rasch model. To determine whether observed differences between subgroups could be explained by non-equivalence of the items, we conducted the DIF analyses. Interestingly, some items showed significant DIF according to the type of hospital and nurse position. Items 7 (patient decision making) and 9 (understanding of medical treatment options) were found to be more difficult among nurses in tertiary hospitals. One possible explanation is that the high disease severity of the hospitals has increased the burden of primary care for the nurses, which makes it difficult to provide enough primary care. As Blakeman, Macdonald, Bower, Gately and Chew-Graham [8] noted, Korean nurses working in hospitals find it difficult to engage in consultations with patients, due to their limited time, even when they perceived patient self-management as important.

Furthermore, item 12 (information seeking) was more difficult for unit managers and specialists compared to staff nurses. Other studies have shown that healthcare providers do not fully utilize health literacy strategies to enhance patients’ understanding of the health information provided [24,25,26]. Despite the essential role of healthcare providers in building and improving patients’ health literacy, many remain unaware or lack knowledge of the relevance of health literacy in patient care.

### Study Limitation

The large sample size was among the strengths of the present study. However, this study has some limitations. First, nurses’ beliefs and attitudes were assessed using a self-reported measure that can be influenced by method biases such as memory recall. Second, the limitations of this study relate to its cross-sectional design, which did not allow for assessment of the test–retest reliability. The sample also indicates that more evidence regarding the responsiveness of the items is needed. Another limitation was that the data were only collected from staff nurses, unit managers, and specialists, and did not include other healthcare providers. Therefore, the findings might not be representative of all healthcare providers. Lastly, to examine the extent to which healthcare providers’ beliefs and attitudes about patient activation influence improve the quality of care, sound cross-culturally equivalent measurements are required. The results of this study illustrate the need to pay attention to these kinds of measurement issues.

## 5. Conclusions

Based on this Rasch analysis, the Korean CS-PAM has good psychometric properties to assess nurses’ beliefs and attitudes toward their support for patient activation. The findings indicate that nurses were the most likely to support aspects of self-management related to a patient making behavior and lifestyle changes in accordance with medical advice, and least likely to support people being independent seekers of information. These competencies are important for patients to successfully self-manage their health and care [23]. Understanding patient–nurse role relationships and their association with activation are essential for ensuring patients’ health and well-being. Therefore, the CS-PAM will be useful in future clinical practice and research to improve the support system and to collaborate with the healthcare team for patient-centered care.

## Figures and Tables

**Table 1:** Demographic characteristics of the sample.

Characteristic	Unit Manager (*n* = 92)	Staff Nurse (*n* = 947)	Specialist (*n* = 30)
Age (years), median (IQR)	47 (3.0)	29 (8.0)	37 (9.0)
20–29	-	504 (53.2)	3 (10.0)
30–39	6 (6.5)	351 (37.1)	17 (56.7)
40–49	57 (62.0)	85 (9.0)	10 (33.3)
50–59	29 (31.5)	7 (0.7)	-
Gender			
Female	91 (98.9)	927 (97.9)	30 (100)
Male	1(1.1)	20 (2.1)	-
Religion			
Yes	50 (54.3)	356 (37.6)	14 (46.7)
No	42 (45.7)	591 (62.4)	16 (53.3)
Marital status			
Married	78 (84.8)	662 (69.9)	9 (30.0)
Not married	14 (15.2)	285 (30.1)	21 (70.0)
Education level			
Associate	18 (19.6)	217 (22.9)	5 (16.7)
Bachelor	6 (6.5)	660 (69.7)	12 (40.0)
Master’s or doctoral	68 (73.9)	70 (7.4)	13 (43.3)
Usual shift			
Nine to five	92 (100)	53 (5.6)	24 (80.0)
Rotating with night shift	-	865 (91.3)	6 (20.0)
Day or evening only	-	29 (3.1)	-
Work tenure (month), median (IQR)	281 (90.25)	64 (92.0)	169.5 (65.25)

IQR = interquartile range. Percentages are based on complete data.

**Table 2:** Item calibration and the infit and outfit statistics for each item in the Korean CS-PAM.

As a Nurse (Clinician), How Important Is it to You that Your Patients with Long Term Conditions	Measure	SEM	Infit MNSQ	Outfit MNSQ
1. Are able to take actions that will help prevent or minimize symptoms associated with their health condition(s).	40.7	0.08	0.99	1.14
2. Are able to figure out solutions when new situations or problems arise with their health condition(s).	44.3	0.07	0.93	0.85
3. Bring a list of questions to their office visit.	61.1	0.06	1.28	1.38
4. Are able to make and maintain lifestyle changes needed to manage their chronic condition.	41.1	0.07	0.92	0.95
5. Can follow through on medical treatments you have told them they need to do at home.	43.8	0.07	0.94	0.93
6. Know what each of their prescribed medications is for.	43.6	0.07	0.91	0.78
7. Are able to determine when they need to go to a medical professional for care and when they can handle the problem on their own.	55.0	0.06	1.12	1.09
8. Understand which of their behaviors make their chronic condition better and which ones make it worse.	41.8	0.07	0.85	0.71
9. Understand the different medical treatment options available for their chronic condition(s).	43.9	0.07	0.82	0.79
10. Tell you the concerns they have about their health even when you do not ask.	56.6	0.06	0.88	0.86
11. Want to be involved as a full partner with me in making decisions about their care.	48.4	0.07	0.87	0.79
12. Look for trustworthy sources of information about their health and health choices, such as on the web, news stories, or books.	63.3	0.06	1.30	1.41
13. Want to know what procedures or treatments they will receive and why before the treatments or procedure are performed.	44.5	0.07	1.07	1.16

Note. Measure = The calibrated scale value of the item; this represents how much activation is required to endorse the item. SEM = standard error of measurement in the estimation of the item difficulty; the SEM is the precision of the item difficulty estimation and ranges from 0 to 100. Infit MNSQ = Infit mean square error, one of two quality control fit statistics assessing item dimensionality (the degree to which the item falls on the same single, real number line as the rest of the items); the infit is an information-weighted residual of the observed responses from the expected responses, and is most sensitive to item fit when the item is located near the person’s scale location. Outfit MNSQ = Outfit mean square error, the fit statistics of which are most sensitive to item dimensionality when the item scale location is distant from the person’s scale location.

## Appendix A

**Table A1:** Comparison to groups classification the CS-PAM by the US, Dutch, and Korea.

Group	US [1]		Dutch [3]		Korea	
	Item	Naming	Item	Naming	Item	Naming
**1**	1,4,8,5	Patient should follow medical advice	1,4,8	Patient should have knowledge and behave in order to prevent or minimize symptoms associated with their health condition	1,4,8	Patient should have knowledge and behave in order to prevent or minimize symptoms associated with their health condition
**2**	2,6,7	Patient can make independent judgments & actions	2,5,6,7,11	Patient can make independent judgments & actions	2,5,6,9,13	Patient can make independent judgments & actions
**3**	9,10,11,13	Patient is able to function as member of care team	9,10,13	Patient is able to take an active role during consultations	7,10,11	Patient is able to function as member of care team
**4**	3,12	Patient should be an independent information seeker	3,12	Patient should be an independent information seeker	3,12	Patient should be an independent information seeker
